# One Syndrome, Many Faces: A Unified Perspective on Heart Failure Phenotypes

**DOI:** 10.3390/ijms26188960

**Published:** 2025-09-15

**Authors:** Ioannis Paraskevaidis, Elias Tsougos, Christos Kourek

**Affiliations:** 1 Medical School of Athens, National and Kapodistrian University of Athens, 15772 Athens, Greece; iparas@otenet.gr; 2 Department of Cardiology, Hygeia Hospital, 15123 Athens, Greece; tsougos@yahoo.com

**Keywords:** heart failure, diastolic dysfunction, preserved ejection fraction, cardiovascular disease, phenotypic classification

## Abstract

Heart failure (HF) remains a major clinical syndrome traditionally classified by left ventricular ejection fraction (EF) into three phenotypes: reduced (HFrEF), mildly reduced (HFmrEF), and preserved (HFpEF). Although EF-based phenotyping has served as a practical framework for diagnosis and treatment stratification, growing evidence challenges its pathophysiological specificity. Clinical trials often blur these categories by including patients with EF > 40% under the HFpEF umbrella, despite current guidelines reserving that range for HFmrEF. This inconsistency introduces ambiguity and undermines the concept of discrete disease entities. In this comprehensive review, we explore the hypothesis that HF is not a group of separate syndromes but rather a single entity manifesting along a spectrum determined by the balance between pathological insult and the patient’s homeostatic adaptive capacity. Emerging data reveal that all HF phenotypes, regardless of EF, share common molecular, cellular, and systemic mechanisms, including neurohormonal activation, inflammation, mitochondrial dysfunction, fibrosis, and programmed cell death. We propose a paradigm shift: from viewing HF through the lens of EF stratification to a unified, mechanistically driven model that recognizes HF as a syndrome with variable manifestations. Reframing HF in this way could enhance diagnostic precision, therapeutic targeting, and research design.

## 1. Introduction: The Complexity of Heart Failure Classification

According to current guidelines [1], heart failure (HF) is defined as a complex clinical syndrome with symptoms and signs resulting from structural or functional impairment of ventricular filling or ejection. Although this definition emphasizes pressure and volume changes throughout the cardiac cycle, clinical practice tends to favor a phenotypic classification based on left ventricular ejection fraction (EF). This categorization includes the following:

(A) EF > 50% (heart failure with preserved EF, HFpEF);
(B) EF between 41–49% (heart failure with mildly reduced EF, HFmrEF);
(C) EF < 40% (heart failure with reduced EF, HFrEF).

However, many studies that claim to focus on HFpEF include patients with EF >40%, thereby incorporating individuals who fall into the HFmrEF category according to current guidelines. To justify this broadened inclusion, elevated levels of N-terminal pro-B-type natriuretic peptide (NT-proBNP) are often used, with cut-offs of <300 pg/mL in sinus rhythm and <600 pg/mL in atrial fibrillation. Notably, NT-proBNP levels can remain within the normal range in up to 20% of patients with HFpEF [2].

Adding further complexity, 10–60% of patients initially classified as HFrEF may later demonstrate improvement in EF, thus transitioning into a new category referred to as heart failure with improved EF (HFimpEF) [3,4]. This fluidity undermines the assumption that EF-based categories are stable and pathophysiologically distinct. EF values can fluctuate due to treatment, disease progression, or measurement variability, making it an unreliable marker for consistent classification over time [5].

Even in expert hands, echocardiographic EF measurements are subject to considerable inter- and intra-observer variability, further complicating the clinical utility of EF as a robust biomarker. Consequently, inconsistencies in classification based on EF may explain why therapies effective in HFrEF or HFmrEF show limited efficacy in HFpEF [6]. Supporting this concern, it has been observed that “the reproducibility of LVEF measurement is poor and its prognostic and diagnostic value lessens when it is above 45%, with no relationship to the severity of cardiac dysfunction or outcomes at higher values” [7].

Thus, the current EF-based phenotypic division may not represent distinct diseases but rather different manifestations of the same syndrome, shaped by the underlying cause and the body’s homeostatic response to varying degrees of insult. In this review, we aim to reassess heart failure not as three separate diseases but as a single syndrome with multiple clinical presentations shaped by diverse underlying mechanisms and differential homeostatic responses.

## 2. HFpEF: A Syndrome or an Epiphenomenon?

Among the inclusion criteria for HFpEF are structural or functional abnormalities consistent with left ventricular (LV) diastolic dysfunction. These typically include concentric LV hypertrophy and left atrial enlargement [8]. However, most patients diagnosed with HFpEF also present with arterial hypertension—a common risk factor coexisting with other comorbidities like obesity, diabetes mellitus, and coronary artery disease [9,10].

This raises a critical question: is the observed diastolic dysfunction a standalone entity, or is it simply the cardiac consequence of longstanding hypertension—essentially hypertensive cardiomyopathy [11]? Indeed, large-scale studies show that arterial hypertension is present in 80–90% of patients enrolled in HFpEF trials [12,13,14,15]. Moreover, while cardiac hypertrophy is often used as an inclusion criterion, it is absent in 40–70% of HFpEF patients, challenging its validity as a defining feature [16,17].

### 2.1. The Role of the ‘Mosaic Theory’ in Hypertension and HF

The mosaic theory of hypertension posits that multiple mechanisms, environmental, neurohormonal, age-related vascular stiffness, oxidative stress, and inflammatory mechanisms, contribute to disease pathogenesis [17]. These mechanisms are also implicated across all HF phenotypes. Therefore, when the majority of HFpEF trial participants are hypertensive, one might argue that HFpEF is more accurately an epiphenomenon of hypertensive cardiomyopathy rather than a distinct syndrome.

### 2.2. Is Left Atrial Enlargement a Diagnostic Clue or a Nonspecific Marker?

The second structural hallmark of HFpEF is left atrial enlargement. This is generally attributed to elevated LV filling pressures; however, other conditions such as paroxysmal atrial fibrillation or mild mitral regurgitation can also cause atrial dilation. In early diastolic dysfunction (stage I), left atrial pressure is typically normal. This raises an important clinical dilemma: is the absence of increased left atrial pressure indicative of the absence of diastolic dysfunction, or does it reflect preserved atrial compliance compensating for elevated LV pressure?

### 2.3. Aging and Diagnostic Ambiguities

Over 70% of patients with HFpEF are older than 65 years [18], suggesting that age-related cardiovascular changes may contribute to the observed phenotype. These findings raise further doubts about whether HFpEF is a true pathologic entity or a manifestation of aging. Although such questions do not refute the existence of HFpEF, they do challenge its current diagnostic framework and suggest its actual prevalence may be significantly lower than the oft-cited 50% of all HF cases [19].

### 2.4. HFpEF as a Systemic Syndrome with Multiple Contributors

Several specific conditions, such as amyloidosis, sarcoidosis, iron overload, and fibrotic disorders, are known to alter myocardial composition, increase filling pressures, and reduce cardiac output. However, these are distinct from the more commonly cited causes of HFpEF, metabolic syndrome and coronary artery disease, which are also prevalent in other HF phenotypes.

As a result, HFpEF appears to be a systemic syndrome involving multiple triggers (e.g., inflammation), contributors (e.g., aging, genetics, gender), and comorbidities (e.g., obesity, diabetes, renal dysfunction) [19]. In this context, six phenotypic subtypes of HFpEF have been proposed:

1. Aging-related;
2. Cardiometabolic;
3. Hypertension-related;
4. Associated with pulmonary arterial hypertension;
5. Coronary artery disease-related;
6. Left atrial myopathy [20].

## 3. Rethinking Heart Failure as a Unified Syndrome

### 3.1. A Common Pathophysiological Foundation

**Figure 1:**
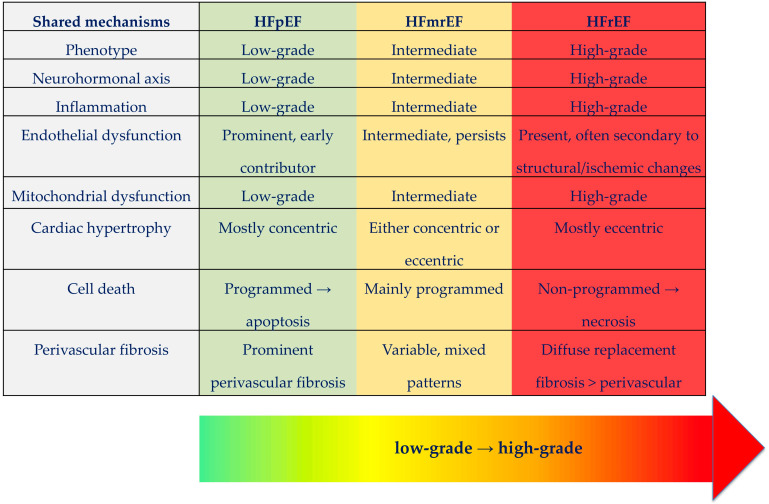
Spectrum of heart failure phenotypes and shared mechanisms. Heart failure presents as a continuum with three main phenotypes: HFrEF (reduced ejection fraction), HFmrEF (mildly reduced ejection fraction), and HFpEF (preserved ejection fraction). Central shared mechanisms across all phenotypes include neurohormonal activation, inflammation, mitochondrial and endothelial dysfunction, hypertrophy, cell death and fibrosis. The phenotypes differ in the intensity of these processes and in the degree of compensatory homeostatic response. HFpEF and early HFmrEF represent low-grade phenotypes, characterized by milder structural changes and subclinical dysfunction, whereas advanced HFmrEF and HFrEF represent high-grade phenotypes with more severe remodeling, cardiomyocyte loss, and decompensation.

HF is characterized by the heart’s inability to meet peripheral metabolic demands under normal filling pressures at rest or during exertion. If we accept this functional definition, it supports the notion that all HF phenotypes, regardless of EF, share a common pathophysiological origin. That is, reduced cardiac output and elevated filling pressures initiate similar compensatory responses: neurohormonal activation, immune signaling, oxidative stress, and energetic insufficiency (Figure 1).

Despite this shared foundation, structural differences among HF phenotypes may reflect the heart’s variable capacity to respond to specific insults. For instance, do conditions such as hypertensive cardiomyopathy and infiltrative diseases (e.g., amyloidosis, sarcoidosis) truly share a unified pathophysiology and homeostatic response? If not, grouping them under a single label, like HFpEF, may cause diagnostic ambiguity.

Indeed, the etiological complexity of HFpEF, which includes microvascular dysfunction, low-grade inflammation, and oxidative stress, raises questions about whether these features are intrinsic to the syndrome or secondary to coexisting comorbidities [20]. While the hemodynamic definition of HF is grounded in objective measurements (e.g., cardiac output, filling pressures), EF-based classification is influenced by subjective symptom assessment and imaging-based volumetric parameters, which are prone to variability [21].

### 3.2. The Limitations of Ejection Fraction: What Is Normal?

There is no universally accepted definition of normal EF. Different studies propose values ranging from 55–70%, while major societies like the American College of Cardiology (ACC)/American Heart Association (AHA) and the European Society of Cardiology (ESC) recommend thresholds above 50%. Interestingly, therapeutic outcomes differ between patients with EF < 57% and those above that threshold, suggesting the current cut-off may not be optimal [13].

Furthermore, EF measurement is technique-dependent. Echocardiography is the most widely used modality, but is subject to significant inter- and intra-observer variability. MRI, though more reliable, is expensive and not readily available in routine practice. This variability can lead to inconsistent patient classification and affect clinical trial outcomes.

In light of these challenges, EF-based subdivision may misrepresent the spectrum of HF and obscure the underlying biology. This warrants a return to core pathophysiologic principles to guide a more integrated classification.

## 4. Pathophysiological Mechanisms of Heart Failure

### 4.1. Disease Progression and Homeostatic Imbalance

HF is a multifactorial syndrome arising from complex anatomical, functional, and biological derangements. These result from a sustained imbalance between external insults and the body’s compensatory mechanisms. Regardless of the initial trigger, the path follows a similar trajectory involving activation of neurohormonal, inflammatory, and oxidative pathways [14].

Not all patients with LV dysfunction develop symptoms, highlighting that impaired EF alone is insufficient to define clinical HF. Differences in disease progression likely reflect heterogeneity in homeostatic responses. Therefore, the phenotypic division of HF into HFrEF, HFmrEF, and HFpEF may represent degrees of severity of a common syndrome, shaped primarily by the magnitude and efficacy of the body’s compensatory response.

Sustained activation of neurohormonal and inflammatory pathways leads to progressive myocardial damage: fibrosis, apoptosis, hypertrophy, arrhythmias, and eventual decompensation [22]. Patients with recovered EF after dilated cardiomyopathy often exhibit persistent neurohormonal activation, supporting the notion that structural recovery may not equate to physiological resolution [23,24,25].

Individual variability in cardiac remodeling, driven by sex, race, genetics, and environmental factors, further complicates the phenotype [22]. Notably, female sex is a known predictor of favorable reverse remodeling, possibly due to estrogen receptor-mediated cellular effects [26]. These examples support the concept that HF phenotype is not dictated solely by etiology but by the dynamic interaction between insult and response.

### 4.2. Neurohormonal Activation Across Phenotypes

One of the earliest and most consistent features of HF is neurohormonal activation involving the renin–angiotensin–aldosterone system (RAAS), the sympathetic nervous system (SNS), and arginine vasopressin (AVP) pathways. These systems are upregulated even in preclinical (stage A) HF and become progressively overexpressed with disease advancement [27,28].

Regardless of EF, this activation contributes to myocardial fibrosis, oxidative stress, hypertrophy, and inflammation [21,28,29]. Neurohormonal dysregulation is reflected in elevated biomarkers such as renin, aldosterone, catecholamines, natriuretic peptides, troponin, C-reactive protein (CRP), interleukins, and markers of extracellular matrix turnover such as galectin-3 and soluble suppression of tumorigenicity 2 protein (ST2). While biomarker levels differ by phenotype, being highest in HFrEF, intermediate in HFmrEF, and lowest in HFpEF, the same pathophysiologic cascade is involved [30].

Notably, 67% of patients with HFpEF exhibit elevation in at least one biomarker, though only 10% have elevations across all markers [29,31]. Moreover, in HFpEF, higher aldosterone and renin levels correlate with worse outcomes, reinforcing the central role of RAAS in myocardial stiffness and prognosis [29,32,33,34].

#### 4.2.1. Sympathetic–Parasympathetic Imbalance

Dysregulation of the autonomic nervous system, with sympathetic overactivity and parasympathetic withdrawal, is implicated in all HF types. Norepinephrine levels rise progressively across the spectrum, highest in HFrEF, intermediate in HFpEF, and lowest in healthy individuals [35]. Elevated norepinephrine correlates with increased pulmonary capillary wedge pressure and poor prognosis, particularly when levels exceed 600 pg/mL [36,37].

Sympathetic overactivity leads to beta-receptor desensitization, impaired contractility, vasoconstriction (via alpha-receptors), and increased afterload. These changes contribute to energetic failure and disease progression [28].

#### 4.2.2. Vasopressin System Dysregulation

The AVP system plays a critical role in fluid and electrolyte homeostasis. Both centrally (hypothalamic) and peripherally (cardiac and pulmonary) derived vasopressin peptides are involved in cardiovascular regulation [38,39]. AVP is activated by hypotension, inflammation, and stress, and interacts with autonomic and inflammatory pathways [39,40,41].

In HF, overactivation of V1a receptors induces vasoconstriction and increased afterload, while V2 receptor stimulation enhances water retention and congestion through sodium and water reabsorption [42,43,44]. Thus, AVP contributes to both hemodynamic and volume overload in all HF phenotypes [45,46].

## 5. Inflammation and Immune Dysregulation

### 5.1. Inflammation as a Shared Pathogenic Process

While inflammation is often emphasized in HFpEF due to its association with metabolic comorbidities (e.g., obesity, diabetes, hypertension), evidence suggests it is equally central in HFrEF pathogenesis [47,48]. According to a consensus from the ESC Working Group on Myocardial Function, structural and functional cardiovascular abnormalities in HF extend beyond comorbidity-driven mechanisms alone [49].

Across all HF phenotypes, systemic inflammation plays a central role, with elevated circulating levels of CRP, interleukin-6 (IL-6), tumor necrosis factor-α (TNF-α), and soluble ST2 consistently reported [50,51]. These biomarkers not only reflect low-grade systemic inflammation but also correlate with disease severity and adverse outcomes, contributing to remodeling and progressive dysfunction.

### 5.2. Sterile Inflammation and Pattern Recognition

A growing body of evidence highlights the pivotal role of sterile inflammation in the pathogenesis of HF, irrespective of phenotype [52]. Unlike pathogen-driven inflammation, sterile inflammation is triggered by endogenous molecules released from injured or stressed cardiomyocytes and vascular cells, known as damage-associated molecular patterns (DAMPs) [53,54]. Examples of DAMPs include high-mobility group box 1 (HMGB1), heat shock proteins, extracellular adenosine triphosphate (ATP), and mitochondrial DNA fragments [55]. These molecules act as “danger signals” and are recognized by pattern recognition receptors (PRRs) expressed on cardiomyocytes, fibroblasts, endothelial cells, and immune cells [56,57].

Among PRRs, Toll-like receptors (TLRs) and NOD-like receptors (NLRs) are particularly relevant to HF [58]. Activation of TLRs, especially TLR2 and TLR4, leads to nuclear factor κB (NF-κB) signaling, promoting the transcription of pro-inflammatory cytokines such as IL-1β, IL-6, and TNF-α [59,60]. Meanwhile, NLRs, notably the NLRP3 inflammasome, act as cytosolic sensors of cellular stress and amplify inflammatory signaling by activating caspase-1 and processing pro-IL-1β into its active form [61]. Persistent engagement of these pathways fuels chronic low-grade inflammation, creating a vicious cycle of leukocyte recruitment, oxidative stress, and maladaptive tissue remodeling.

Functionally, this sterile inflammation drives fibrosis, endothelial dysfunction, and cardiomyocyte death, thereby impairing both systolic and diastolic function. Although HF phenotypes differ in their structural and clinical presentation, sterile inflammation represents a common denominator that sustains disease progression. In HFpEF, PRR activation is often comorbidity-driven (e.g., obesity, hypertension), while in HFrEF, it is closely linked to ischemia and cardiomyocyte necrosis [62]. Thus, PRR-mediated sterile inflammation provides a unifying mechanistic framework that contributes to adverse outcomes across the HF spectrum.

### 5.3. Immune Response and Comorbidity-Driven Inflammation

In HFpEF, protein misfolding, oxidative stress, and mitochondrial dysfunction trigger distinct transcriptomic and immunologic profiles compared to HFrEF [62]. The degree of immune activation is modulated by the nature and severity of the underlying insult: for example, myocardial infarction elicits robust inflammatory responses aimed at clearing necrotic tissue, whereas metabolic syndromes promote low-grade para-inflammation. Although innate and adaptive immune responses are activated in both HFpEF and HFrEF, they differ in intensity and regulation. In HFpEF, systemic comorbidities such as obesity, hypertension, and diabetes drive chronic low-grade inflammation characterized by endothelial dysfunction, leukocyte adhesion, and T-cell infiltration [63,64,65,66]. By contrast, HFrEF is strongly associated with ischemic injury and cardiomyocyte necrosis, which release DAMPs and trigger heightened innate immune activation, including Toll-like receptor signaling, complement activation, and recruitment of neutrophils and monocytes to sites of injury [65]. On the adaptive side, HFrEF is marked by pronounced B-cell and T-cell dysregulation, with evidence of autoreactive antibodies, cytotoxic T-cell responses against cardiomyocytes, and reduced regulatory T-cell activity [67,68]. Collectively, these processes sustain persistent inflammation, adverse remodeling, and progressive systolic dysfunction. Thus, both HF phenotypes involve similar immunoinflammatory mechanisms but differ in timing and magnitude: HFrEF typically reflects an acute inflammatory burst followed by chronic persistence, while HFpEF predominantly manifests as a chronic, low-grade inflammatory state. Together, they represent variations of the same fundamental process.

## 6. Myocardial Cellular Changes in Heart Failure

### 6.1. Fibrosis and Cellular Remodeling

One fundamental difference between HF phenotypes lies in the degree and nature of myocardial fibrosis. As noted, “differences in the localization, composition, and crosslinking of the cardiac fibrous tissue contribute to the differences in HFrEF and HFpEF” [69]. While myofibroblast activation follows similar pathways in both HFpEF and HFrEF, the downstream signals, including matrix metalloproteinases (MMPs), tissue inhibitors of MMPs (TIMPs), and their ratios, interact differently with fibrogenic mediators such as TGF-β1, plasmin, MMP-9, and integrins. These molecular interactions influence fibrosis-related gene expression in distinct ways [70].

It is worth noting that histological evidence of myocardial fibrosis in HFpEF is relatively limited. Autopsy studies often report only mild fibrosis without significant differences in collagen volume fraction between HFpEF and control hearts [71]. Moderate-to-severe fibrosis is observed in only 26% of HFpEF patients [72].

### 6.2. Patterns of Myocardial Cell Death

**Figure 2:**
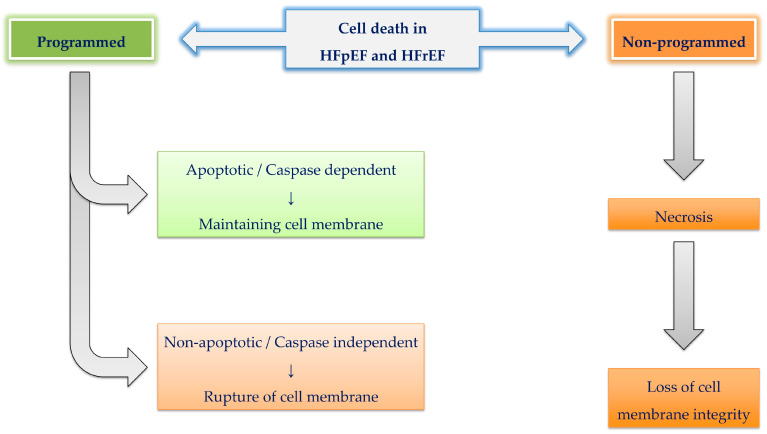
Programmed and non-programmed cell death in heart failure phenotypes. Cell death in heart failure occurs through non-programmed (necrosis) and programmed pathways. Programmed pathways are subdivided into apoptotic/caspase-dependent (apoptosis) and non-apoptotic/caspase-independent mechanisms (including necroptosis, pyroptosis, ferroptosis, autophagy, and mitoptosis). HFrEF is predominantly characterized by necrosis and extensive membrane disruption, whereas HFpEF more often involves programmed forms of cell death. Both phenotypes, however, share overlapping molecular death mechanisms.

Another key anatomical distinction between HF phenotypes is the mechanism of cardiomyocyte loss. Cell death occurs via necrosis (predominant in HFrEF?) or apoptosis (more frequent in HFpEF?), with necrosis involving cell swelling and rupture, and apoptosis characterized by cytoplasmic and nuclear shrinkage (Figure 2). Elevated troponin levels in HFrEF support a greater extent of cardiomyocyte injury in this phenotype [69].

Current understanding divides cell death into non-programmed (e.g., necrosis) and programmed pathways, with the latter further subdivided into apoptotic and non-apoptotic processes [73]. Although data on cell death mechanisms in HFpEF remain sparse, emerging evidence indicates that both phenotypes involve overlapping modes of cell death, including apoptosis, necroptosis, autophagy, mitoptosis, ferroptosis, and pyroptosis [69,74,75].

Interestingly, programmed non-apoptotic pathways, proposed to dominate in HFpEF, can still lead to membrane rupture akin to necrosis, commonly associated with HFrEF [76]. Conversely, low levels of apoptosis are also observed in HFrEF. Thus, both forms of HF likely share common molecular death mechanisms, and it is the severity of the insult and the homeostatic response that shape their phenotypic expression. Notably, both necrosis and apoptosis affect mitochondria, causing either swelling or shrinkage, resulting in varying degrees of mitochondrial content leakage.

## 7. Mitochondrial Dysfunction and Oxidative Stress

### 7.1. Central Role of Mitochondria

Mitochondria are abundant in cardiomyocytes and sustain essential functions, including ATP generation, calcium regulation, redox balance, and control of cell death [77]. In heart failure, mitochondrial dysfunction leads to energy depletion, which in turn contributes to ATP-dependent cell death via apoptosis or necrosis depending on ATP availability, cell type, and environmental context [78]. Beyond energy failure, dysfunctional mitochondria generate excessive reactive oxygen species (ROS), promote inflammatory responses, impair calcium cycling, and alter gene expression programs linked to programmed cell death [78]. These abnormalities are observed across HF phenotypes and are exacerbated by neurohormonal overactivation and impaired metabolic flexibility, resulting in disturbed ATP/adenosine diphosphate (ADP)/phosphocreatine (PCr) ratios and energetic starvation in both preserved and reduced EF, as well as in left and right ventricular failure [79]. Furthermore, comorbidities such as diabetes, obesity, and hypertrophy aggravate metabolic remodeling, disrupting PCr/ATP balance and correlating with more advanced HF symptoms and worse NYHA class [80].

Mitochondrial dynamics also play a key role in cardiomyocyte homeostasis. Fission, mediated by dynamin-related protein 1 (Drp1), is essential for removing damaged mitochondrial fragments but becomes excessive in HF, causing mitochondrial fragmentation, loss of membrane potential, and increased apoptotic susceptibility [81,82]. Fusion, controlled by mitofusins (Mfn1/2) and optic atrophy protein 1 (Opa1), maintains mitochondrial integrity and dilutes localized damage, yet impaired fusion in both HFpEF and HFrEF contributes to energetic inefficiency and oxidative stress [83,84,85]. Mitophagy, the selective clearance of dysfunctional mitochondria, is an adaptive process, but both excessive and insufficient mitophagy have been described in HF, linking altered quality control to progressive dysfunction and cell death [86].

Importantly, the severity and nature of these alterations differ between phenotypes. HFpEF typically shows subtle impairments in mitochondrial dynamics consistent with low-grade energetic stress [87], whereas HFrEF demonstrates more severe abnormalities, including marked fission activation, reduced fusion, and defective mitophagy [88]. Collectively, these disturbances create a vicious cycle of bioenergetic failure and oxidative damage that fuels adverse remodeling and disease progression across the HF spectrum.

### 7.2. Mitochondrial Defense and Organelle Crosstalk

Mitochondrial quality control mechanisms, such as fission, fusion, and mitophagy, are activated across HF phenotypes. These are more effective in earlier or milder disease stages (e.g., HFpEF) and progressively overwhelmed in advanced HF (e.g., HFrEF) [89,90]. The nature of mitochondrial stress differs between phenotypes and may influence therapeutic strategies [91,92]. In HFpEF, mitochondrial stress is primarily characterized by impaired oxidative phosphorylation efficiency, increased production of ROS, and reduced metabolic flexibility, often in the context of comorbidity-driven microvascular dysfunction [93,94]. These alterations create a chronic but relatively low-grade energetic deficit. In HFrEF, mitochondrial stress is more profound and often driven by ischemic injury, excessive mitochondrial fission, and impaired mitophagy, leading to widespread mitochondrial loss, ATP depletion, and heightened susceptibility to necrosis [89]. Consequently, therapeutic strategies may need to be tailored: in HFpEF, interventions that improve mitochondrial efficiency, enhance nitric oxide bioavailability, or restore metabolic flexibility (e.g., exercise, SGLT2 inhibitors, mitochondrial-targeted antioxidants) may prove beneficial [87,95,96,97]. In HFrEF, therapies directed at reducing excessive fission, stimulating mitochondrial biogenesis, or restoring mitophagy and energy production including Drp1 inhibitors, peroxisome proliferator-activated receptor gamma coactivator-1α (PGC-1α) activators, or mitophagy enhancers, may be more appropriate [91,98]. These differences suggest that phenotype-specific targeting of mitochondrial pathways could represent a promising avenue for precision therapy in HF.

Importantly, effective crosstalk between mitochondria and the endoplasmic reticulum (ER) is vital for homeostasis. Disruption of this communication results in protein misfolding, excessive ROS production, calcium dysregulation, and activation of inflammatory and apoptotic pathways, all of which contribute to HF pathogenesis and progression [99,100,101].

### 7.3. Reactive Oxygen Species and Oxidative Stress

ROS, primarily generated by mitochondria (mtROS), have essential signaling roles but become pathogenic when produced in excess, overwhelming mitochondrial antioxidant defenses and disrupting redox balance. This imbalance causes damage to proteins, lipids, and nucleic acids, leading to inflammation, energetic failure, and structural remodeling. The severity and duration of oxidative stress modulate HF phenotype: HFrEF is typically characterized by higher levels of mtROS and more severe oxidative injury, while HFpEF exhibits comparatively lower mtROS production, often originating from endothelial dysfunction rather than direct cardiomyocyte injury [93,102]. Importantly, recent studies emphasize that the efficiency of defense mechanisms, rather than the precise ROS source, plays a decisive role in determining outcomes [103,104,105]. Several molecular pathways contribute to oxidative stress–mediated damage across HF phenotypes, including AMP-activated protein kinase (AMPK) signaling [106], mitochondrial and cytosolic antioxidant systems [107], the cyclic guanosine monophosphate (cGMP)–protein kinase G (PKG) pathway [108], and MMPs regulation [105]. While these mechanisms are universally activated, their intensity is greater in HFrEF, reflecting the phenotype’s more profound oxidative burden.

## 8. Molecular Pathways in HF (AMPK, MMP, cGMP–PKG)

Although common molecular pathways underlie all HF phenotypes, their relative activation and downstream consequences differ substantially, with important therapeutic implications. AMPK signaling represents a central metabolic sensor that regulates energy homeostasis, fatty acid oxidation, and glucose uptake. In HFpEF, AMPK activity is frequently suppressed due to the burden of metabolic comorbidities such as obesity, diabetes, and hypertension, which impair substrate flexibility and limit the myocardium’s ability to adapt to stress [77,109,110]. By contrast, in HFrEF, AMPK may be partially activated in response to energy depletion caused by impaired oxidative phosphorylation and mitochondrial dysfunction [77,111]. This differential regulation suggests that AMPK activators may hold greater promise in HFpEF, where restoring energy sensing could correct metabolic inflexibility, while in HFrEF the benefits may be more modest and supportive rather than disease-modifying.

Similarly, MMPs play divergent roles across phenotypes. In HFrEF, robust upregulation of MMPs contributes to extracellular matrix degradation, adverse remodeling, and ventricular dilation, whereas in HFpEF, the process is characterized by subtler fibrotic remodeling and a different balance between MMPs and their tissue inhibitors TIMPs [112,113,114]. Thus, interventions targeting MMP activity, such as selective MMP inhibitors or modulation through angiotensin receptor–neprilysin inhibitors (ARNIs), may be more impactful in HFrEF, while in HFpEF, antifibrotic strategies may need to address the broader inflammatory–fibrotic milieu rather than MMP activity alone [115,116,117].

The cGMP–PKG pathway further illustrates phenotype-specific distinctions. In HFpEF, endothelial dysfunction, impaired nitric oxide bioavailability, and microvascular rarefaction contribute to diminished cGMP generation and reduced PKG activity, which exacerbate diastolic stiffness and impaired relaxation [118,119]. On the other hand, in HFrEF, natriuretic peptide–mediated cGMP production may remain relatively preserved, though insufficient to fully counteract progressive remodeling [120,121]. This divergence implies that sGC stimulators or phosphodiesterase-5 inhibitors may be particularly beneficial in HFpEF, whereas in HFrEF therapies that augment natriuretic peptide signaling, such as ARNIs, may provide greater benefit [122,123]. Collectively, these examples underscore that while HF can be conceptualized as a unified syndrome, precision medicine approaches that integrate phenotype-specific molecular profiles are essential for therapeutic stratification.

Differential activation of selected molecular pathways and therapeutic implications between HFpEF and HFrEF are demonstrated in Table 1.

## 9. Advanced Imaging and Biomarkers in Unifying Heart Failure Diagnosis

Over the past decade, significant advances in cardiovascular imaging and biomarker science have reshaped the understanding of HF phenotypes. Traditional reliance on LVEF alone has been supplemented by a spectrum of diagnostic modalities capable of detecting subtle myocardial changes before overt systolic dysfunction occurs. Cardiac magnetic resonance (CMR) imaging, particularly T1 mapping and extracellular volume (ECV) quantification, allows for the detection of diffuse myocardial fibrosis, a feature common to both HFpEF and HFrEF [124]. Studies have demonstrated that elevated native T1 and ECV values correlate with adverse outcomes regardless of EF category, supporting the concept of HF as a continuous pathophysiological process rather than distinct diseases [125,126].

Echocardiographic strain imaging, especially global longitudinal strain (GLS), has emerged as a sensitive measure of subclinical myocardial dysfunction. Reductions in GLS are found in HFpEF, HFmrEF, and HFrEF, with the degree of impairment correlating with symptom severity and prognosis [127,128]. Notably, GLS abnormalities often precede LVEF reduction, challenging the EF-based staging of HF.

In parallel, circulating biomarkers have reinforced the biological continuum of HF. Natriuretic peptides such as NT-proBNP remain cornerstones in diagnosis, but novel biomarkers, including galectin-3, soluble ST2, high-sensitivity troponins, and growth differentiation factor-15 (GDF-15), provide complementary prognostic information. Importantly, these biomarkers reflect overlapping mechanisms such as fibrosis, inflammation, and cardiomyocyte injury across EF phenotypes [129,130]. Integrating imaging and biomarker data into clinical assessment may thus facilitate a mechanism-based rather than EF-based HF classification. The most significant diagnostic or prognostic biomarkers in HF are presented in Table 2.

## 10. The Role of Comorbidities in the HF Phenotype Spectrum

Comorbidities exert a profound influence on the development, manifestation, and progression of HF. Rather than merely coexisting with HF, conditions such as hypertension, diabetes mellitus, obesity, chronic kidney disease, and atrial fibrillation actively contribute to myocardial remodeling and dysfunction [131,132]. The “comorbidity-driven” paradigm, particularly relevant in HFpEF, posits that systemic inflammation, endothelial dysfunction, and microvascular rarefaction are primary drivers of myocardial stiffening and impaired relaxation [103,133].

Hypertension, the most prevalent comorbidity in HFpEF, induces concentric left ventricular hypertrophy and increases passive stiffness, while promoting microvascular damage [134,135]. Similarly, obesity is associated with increased plasma volume, adipose tissue inflammation, and impaired mitochondrial energetics, contributing to both preserved and reduced EF phenotypes [136,137,138]. Diabetes mellitus accelerates myocardial fibrosis and impairs calcium handling, mechanisms that are not exclusive to any single EF category [139,140]. Chronic kidney disease introduces uremic toxins and volume overload, aggravating both systolic and diastolic dysfunction [141,142].

Interestingly, atrial fibrillation not only complicates the hemodynamics of HF but also serves as a trigger for decompensation in both HFpEF and HFrEF [143]. The high prevalence of multiple comorbidities in individual patients blurs the boundaries between phenotypes, suggesting that EF-based classification inadequately reflects the systemic nature of the syndrome.

Epidemiological data from large registries reveal that the number and severity of comorbidities predict adverse outcomes more strongly than EF [144,145]. This supports a model where HF phenotypes represent different clinical expressions of a single syndrome modulated by comorbidity burden and the balance between injury and homeostatic repair capacity.

## 11. Therapeutic Convergence: Are We Moving Towards a Universal HF Treatment Model?

Historically, pharmacological treatment strategies for HF have been EF-dependent, with the strongest evidence base supporting neurohormonal blockade in HFrEF. However, recent randomized controlled trials have begun to challenge this paradigm, revealing benefits of several therapeutic classes across the EF spectrum.

Sodium-glucose cotransporter-2 (SGLT2) inhibitors, such as dapagliflozin and empagliflozin, have demonstrated consistent reductions in HF hospitalizations and cardiovascular death in patients with HFpEF, HFmrEF, and HFrEF [146,147]. The DELIVER [15] and EMPEROR-Preserved trials [14] expanded the therapeutic landscape for HFpEF, with subgroup analyses showing benefits independent of baseline EF. Similarly, ARNIs have shown efficacy in HFmrEF and some subgroups of HFpEF, particularly those with EF closer to the reduced range [148].

Mineralocorticoid receptor antagonists (MRAs) and β-blockers, once considered primarily HFrEF therapies, also exhibit outcome benefits in patients with mid-range and preserved EF when selected based on biomarker elevation or high-risk clinical profiles [123]. These findings support a shift from EF-based therapy restriction toward mechanism-based treatment allocation, where interventions target common pathophysiological pathways such as neurohormonal activation, inflammation, and fibrosis.

**Figure 3:**
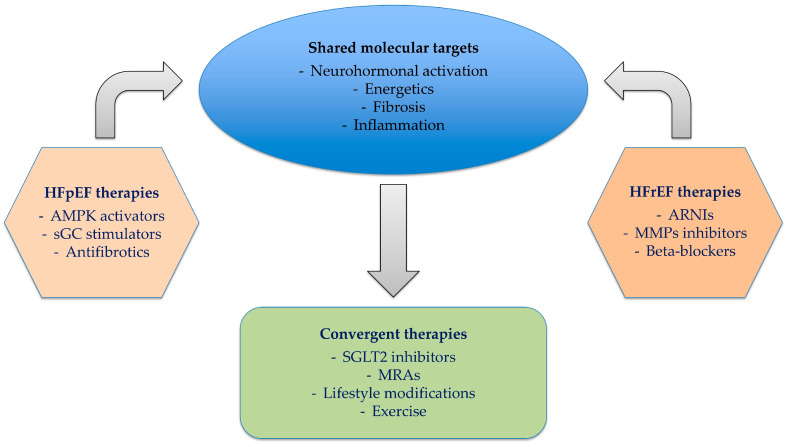
Convergence of therapies and molecular targets among HF phenotypes.

Therapies and their molecular mechanisms among HF phenotypes are shown in Table 3 and Figure 3.

### 11.1. Limitations and Gaps in Literature

Despite the growing body of evidence on HF phenotypes, several limitations and gaps remain. First, much of the current literature relies on EF-based categorization, which, although practical, may oversimplify the underlying biological complexity and mask important mechanistic overlaps between HF phenotypes. Many studies, especially in HFpEF, enroll heterogeneous populations, sometimes including patients with EF between 40–50%, leading to inconsistent results and reduced comparability across trials. Additionally, most mechanistic insights stem from cross-sectional or observational studies, limiting causal inference. There is also a scarcity of longitudinal studies integrating multi-omics, advanced imaging, and functional data to unravel the dynamic progression between phenotypes. The interplay of comorbidities, systemic inflammation, and microvascular dysfunction remains incompletely characterized, particularly in HFpEF. Finally, therapeutic trials often fail to stratify participants based on molecular or pathophysiological subtypes, which may explain the modest benefits observed in some phenotypes. Addressing these gaps requires harmonized definitions, phenotype-specific biomarkers, and personalized intervention strategies supported by robust multicenter trials.

### 11.2. Future Perspectives

Future research on heart failure should move beyond the traditional EF-based classification toward an integrated, multidimensional framework that incorporates molecular signatures, imaging biomarkers, and functional assessments. Advances in CMR, strain imaging, and artificial intelligence-driven analytics offer opportunities to detect early subclinical changes and track phenotypic transitions over time [149,150]. Multi-omics approaches, including genomics, transcriptomics, proteomics, and metabolomics, could help identify patient-specific pathways that drive disease progression, enabling precision medicine strategies [151]. Furthermore, understanding the bidirectional relationship between comorbidities and HF phenotypes may reveal novel preventive and therapeutic targets. Clinical trials should aim for more refined patient selection, incorporating pathophysiological endotypes rather than relying solely on EF thresholds. Finally, translational studies bridging experimental models with human cohorts will be essential for validating mechanistic hypotheses and accelerating therapeutic innovation. By embracing this integrative approach, the field can move closer to tailored interventions that improve outcomes across the HF spectrum.

The convergence of therapeutic evidence across EF categories suggests that HF may be more effectively managed by identifying and targeting dominant biological drivers rather than rigidly adhering to EF cut-offs. This approach aligns with the concept of HF as a single syndrome with variable phenotypic expression, encouraging more personalized and inclusive treatment strategies.

## 12. Conclusions

The traditional phenotypic classification of HF, though valuable for guiding clinical management, may no longer fully reflect the underlying biology of the syndrome. Growing evidence supports the view that all forms of HF, whether reduced, mid-range, or preserved ejection fraction, share common pathophysiological mechanisms. The apparent differences between phenotypes arise primarily from the nature of the initiating insult and the variability of the human homeostatic response.

In more severe or prolonged disease states, the body’s compensatory mechanisms respond differently, leading to diverse clinical manifestations. However, these differences represent varying expressions of a single, complex syndrome rather than distinct disease entities. Reframing HF as a unified syndrome with multiple faces encourages a more integrative approach to diagnosis, research, and treatment.

## Figures and Tables

**Table 1:** Differential activation of selected molecular pathways and therapeutic implications between HFpEF and HFrEF.

Pathway/ Mechanism	HFpEF	HFrEF	Therapeutic Implications
AMPK signaling	Suppressed due to metabolic comorbidities (obesity, diabetes, hypertension) → impaired substrate flexibility	Partially activated as compensatory response to energy depletion	AMPK activators may restore metabolic efficiency in HFpEF; supportive role in HFrEF
MMPs activity	Subtle upregulation; altered MMP/TIMP balance; promotes fibrosis	Strongly upregulated; drives ECM degradation, adverse remodeling, dilation	MMPs inhibitors/ARNIs more effective in HFrEF; antifibrotic strategies needed in HFpEF
cGMP–PKG signaling	Impaired due to endothelial dysfunction, reduced NO bioavailability, microvascular rarefaction	Relatively preserved via natriuretic peptide–driven cGMP, but insufficient to fully counter remodeling	sGC stimulators or PDE5 inhibitors promising in HFpEF; ARNIs beneficial in HFrEF

AMPK, AMP-activated protein kinase; MMP, matrix metalloproteinases; cGMP, cyclic guanosine monophosphate; PKG, protein kinase G; HFpEF, heart failure with preserved ejection fraction; HFrEF, heart failure with reduced ejection fraction; TIMP, tissue inhibitor of metalloproteinases; ECM, extracellular matrix; ARNIs, angiotensin receptor–neprilysin inhibitors; NO, nitric oxide; sGC, soluble guanylate cyclase; PDE5, phosphodiesterase type 5.

**Table 2:** Biomarkers in HF phenotypes: shared and differential patterns.

Biomarker	Pathophysiological Role in HF	Expression Across Phenotypes	Clinical Relevance
C-reactive protein (CRP)	Marker of systemic inflammation	Elevated in HFpEF, HFmrEF, HFrEF	Prognostic of adverse outcomes, reflects low-grade systemic inflammation
Interleukin-6 (IL-6)	Pro-inflammatory cytokine	Increased in all phenotypes, higher in advanced HFrEF	Associated with remodeling, progression, and mortality
Tumor necrosis factor-α (TNF-α)	Cytokine driving apoptosis, cachexia, remodeling	Prominent in HFrEF; also elevated in HFpEF	Linked to cachexia, systolic dysfunction, poor prognosis
Soluble ST2 (sST2)	Biomarker of myocardial stress and fibrosis	Elevated in all phenotypes	Strong prognostic marker, guides risk stratification
NT-proBNP/BNP	Reflects wall stress	Elevated in all phenotypes, higher in HFrEF	Widely used diagnostic/prognostic tool
Troponins (hs-cTnI/T)	Indicator of ongoing myocardial injury	Elevated in HFrEF and myocardial injury; lower but detectable in HFpEF	Predicts adverse events, reflects ongoing cell damage
Galectin-3	Marker of fibrosis and inflammation	Higher in HFpEF	Predicts adverse remodeling and outcomes

HFmrEF, heart failure with mildly reduced ejection fraction; HFpEF, heart failure with preserved ejection fraction; HFrEF, heart failure with reduced ejection fraction.

**Table 3:** Convergence of therapies and molecular targets across HF phenotypes.

Therapy	Primary Target/ Mechanism	HFpEF	HFrEF	Convergence/ Divergence
ARNI (sacubitril/valsartan)	Neurohormonal modulation, natriuretic peptides	Limited benefit (selected patients)	Strong evidence, improves survival	Unified neurohormonal pathway, stronger in HFrEF
SGLT2 inhibitors	Metabolic modulation, improved energetics, anti-inflammatory	Strong evidence for reduced hospitalization	Strong evidence for mortality and hospitalization reduction	Convergent benefit across phenotypes
Beta-blockers	Sympathetic inhibition	Symptom control, limited outcome benefit	Clear mortality benefit	More effective in HFrEF
Mineralocorticoid receptor antagonists (MRAs)	Aldosterone inhibition, antifibrotic	Mixed results	Strong survival benefit	Divergent effect strength
sGC stimulators (vericiguat, riociguat)	Enhance cGMP–PKG signaling	Promising in HFpEF with endothelial dysfunction	Moderate evidence in HFrEF	Phenotype-guided targeting
AMPK activators/metabolic modulators	Restore energy homeostasis, improve substrate use	Strong rationale, ongoing trials	Supportive role	More phenotype-specific for HFpEF
MMP inhibitors/antifibrotics	Extracellular matrix regulation	Potential to limit fibrosis	Potential to limit remodeling	Pathway present in both, but dominant in HFrEF

HFpEF, heart failure with preserved ejection fraction; HFrEF, heart failure with reduced ejection fraction; ARNI, angiotensin receptor–neprilysin inhibitors; sGC, soluble guanylate cyclase; AMPK, AMP-activated protein kinase; MMP, matrix metalloproteinases.

## Abbreviations

The following abbreviations are used in this manuscript:

ACC	American College of Cardiology
ADP	Adenosine Diphosphate
AHA	American Heart Association
AMPK	AMP-Activated Protein Kinase
ARNIs	Angiotensin Receptor–Neprilysin Inhibitors
ATP	Adenosine Triphosphate
AVP	Arginine Vasopressin
cGMP	Cyclic Guanosine Monophosphate
CMR	Cardiac Magnetic Resonance
CRP	C Reactive Protein
DAMPs	Damage-Associated Molecular Patterns
Drp1	Dynamin-Related Protein 1
ECM	Extracellular Matrix
ECV	Extracellular Volume
EF	Ejection Fraction
ER	Endoplasmic Reticulum
ESC	European Society of Cardiology
GDF-15	Growth Differentiation Factor-15
GLS	Global Longitudinal Strain
HF	Heart Failure
HFmrEF	Heart Failure with Mildly Reduced Ejection Fraction
HFpEF	Heart Failure with Preserved Ejection Fraction
HFrEF	Heart Failure with Reduced Ejection Fraction
HMGB1	High-Mobility Group Box 1
IL-6	Interleukin-6
LV	Left Ventricle/Left Ventricular
Mfn1/2	Mitofusins 1/2
MMPs	Matrix Metalloproteinases
MRAs	Mineralocorticoid Receptor Antagonists
mtROS	Mitochondrial Reactive Oxygen Species
NF-κB	Nuclear Factor κB
NLRs	NOD-Like Receptors
NO	Nitric Oxide
NT-proBNP	N-terminal pro-B-type Natriuretic Peptide
NYHA	New York Heart Association
Opa1	Optic Atrophy Protein 1
PCr	Phosphocreatine
PDE5	Phosphodiesterase Type 5
PGC-1α	Peroxisome Proliferator-Activated Receptor Gamma Coactivator-1α
PKG	Protein Kinase G
PRRs	Pattern Recognition Receptors
RAAS	Renin–Angiotensin–Aldosterone System
ROS	Reactive Oxygen Species
sGC	Soluble Guanylate Cyclase
SGLT2	Sodium-Glucose Cotransporter-2
SNS	Sympathetic Nervous System
ST2	Suppression of Tumorigenicity 2 Protein
TGF-β1	Transforming Growth Factor Beta 1
TIMPs	Tissue Inhibitor of Metalloproteinases
TLRs	Toll-Like Receptors
TNF-α	Tumor Necrosis Factor-α
